# Serum Concentration of HDL Particles Predicts Mortality in Acute Heart Failure Patients

**DOI:** 10.1038/srep46642

**Published:** 2017-04-18

**Authors:** Ines Potočnjak, Vesna Degoricija, Matias Trbušić, Gudrun Pregartner, Andrea Berghold, Gunther Marsche, Saša Frank

**Affiliations:** 1 University Hospital Centre Sisters of Charity, Department of Medicine, Zagreb, Croatia; 2 University of Zagreb School of Medicine, Zagreb, Croatia; 3 Institute for Medical Informatics, Statistics und Documentation, Medical University of Graz, Graz, Austria; 4 Institute of Experimental and Clinical Pharmacology, Medical University of Graz, Austria; 5 BioTechMed-Graz, Austria; 6 Institute of Molecular Biology and Biochemistry, Centre of Molecular Medicine, Medical University Graz, Graz, Austria

## Abstract

Clinical studies have shown that assessing circulating concentrations of high-density lipoprotein (HDL) particles by nuclear magnetic resonance (NMR) spectroscopy is superior to HDL-cholesterol in predicting cardiovascular risk. We tested the hypothesis that circulating concentrations of HDL particles predict 3-month mortality of patients with acute heart failure (AHF). Out of 152 included patients, 52% were female, additionally the mean patient age was 75.2 ± 10.3 years, and three-month mortality was 27%. Serum lipoprotein profile at admission was determined by NMR spectroscopy. Univariate logistic regression analyses revealed a significant inverse association of total (odds ratio (OR) 0.38 per 1-SD increase, 95% confidence interval (CI) 0.23–0.60, p < 0.001) and small HDL particle concentrations (OR 0.35 per 1-SD increase, 95% CI 0.19–0.60, p < 0.001) with 3-month mortality, whereas concentrations of large HDL particles (p = 0.353) or HDL-cholesterol (p = 0.107) showed no significant association. After adjustment for age, sex, mean arterial pressure, low-density lipoprotein cholesterol, glomerular filtration rate, urea, and N-terminal pro-brain natriuretic peptide, both the total and small HDL particle concentrations remained significantly associated with 3-month mortality. Based on our results, we conclude that total and small HDL particle concentrations strongly and independently predict 3-month mortality in AHF patients.

## Introduction

Heart failure (HF) remains an important cause of morbidity and mortality worldwide^1^. Accurate prognostic biomarkers are crucial for risk assessment, timely and appropriate therapeutic intervention, and overall management of HF. Therefore, identification of new markers reflecting different aspects of the underlying pathophysiology may improve risk assessment in HF.

High-density lipoprotein (HDL) exerts numerous beneficial effects on the cardiovascular system. These include attenuation of the inflammatory response in the vascular endothelium and macrophages, low-density lipoprotein (LDL) protection from oxidation, endothelial nitric oxide (NO) production stimulation and the associated vasodilatation promotion, as well as insulin-independent glucose uptake stimulation in cardiomyocytes^2^,^3^,^4^,^5^,^6^.

The promotion of reverse cholesterol transport is the best-studied protective activity of HDL. It is a dynamic process by which cholesterol is transported by HDL from the periphery to the liver for excretion^7^. Recent studies delivered solid evidence that cholesterol efflux capacity is inversely related to incident coronary heart events in the general population, independent of well-known cardiovascular risk factors^8^,^9^. However, it has to be pointed out that this association is not present in patients with chronic kidney disease^10^.

Even though clinical and epidemiological studies revealed an inverse relationship between HDL-cholesterol and cardiovascular disease^11^, a number of studies argue against HDL-cholesterol as therapeutic target. This thesis was exemplified by a failure of the Mendelian randomization approach to show a relationship between genetic variants that increase plasma concentrations of HDL cholesterol and a decreased risk of cardiovascular events^12^ as well as by a failure of pharmacological HDL-cholesterol raising therapy to reduce cardiovascular events^13^,^14^. HDL-cholesterol concentrations, therefore, appear to provide limited information regarding the cardioprotective activities of HDL, despite increasing evidence supporting the clinical significance of these pleiotropic HDL functions.

Decreased HDL cholesterol plasma levels and impaired HDL function have previously been reported in HF patients^15^, and several studies examined the association of outcome and prognosis in HF with HDL-cholesterol^16^,^17^,^18^.

Clinical studies have shown that assessing circulating concentrations of HDL particles by nuclear magnetic resonance (NMR) spectroscopy is superior to HDL-cholesterol in predicting cardiovascular risk^19^. HDL particle concentrations attribute equal weight to all HDL subclasses and therefore better represent the biological relationship between HDL and clinical risk^19^. While assessing the functional properties of HDL may not be readily applicable in the everyday clinical practice, NMR-based measurements of HDL particle concentrations bear a great potential for improved assessment of cardiovascular risk.

We hypothesized that HDL particle concentration might predict mortality in subjects presenting with acute heart failure (AHF). Therefore, we explored the association of HDL particle concentrations with 3-month mortality in patients with AHF.

## Results

### Patients

The patients’ baseline characteristics were reported elsewhere^20^. In brief, out of 152 included patients 52% were female, 7.2% belonged to New York Heart Association Functional Classification (NYHA) class 2, 54.6% belonged to NYHA class 3, and 38.2% belonged to NYHA class 4. The mean patient age was 75.2 ± 10.3 years. Frequent comorbidities were hypertension (89.5%), followed by type 2 diabetes mellitus (51.7%), hyperlipidemia/hypertriglyceridemia (39.5%), and hypercholesterolemia (38.8%). Worsening of chronic HF emerged in 69.1% of patients, and 42.4% had a preserved ejection fraction. Three-month mortality was 27.4%. The patients’ laboratory parameters are shown in Table 1.

### Correlation of HDL particle- and HDL cholesterol- concentrations with laboratory and clinical variables

As presented in Table 2, the concentrations of total and small HDL particles were positively correlated with albumin and glomerular filtration rate (GFR) and negatively correlated with urea, N-terminal pro-Brain Natriuretic Peptide (NT-proBNP), and C-reactive protein (CRP). Concentrations of large HDL particles were not significantly correlated with any laboratory parameters, whereas levels of HDL-cholesterol were positively correlated with GFR and negatively correlated with urea and CRP. The concentrations of large HDL particles were negatively correlated with body weight and body mass index (BMI), a correlation that was observed neither for the concentrations of total and small HDL particles nor for HDL- cholesterol (Table 2).

### Logistic regression analyses

Univariate analyses revealed a highly significant inverse association of 3-month mortality with total and small HDL particle concentrations as well as with total cholesterol, LDL-cholesterol, mean arterial pressure (MAP), and GFR (Table 3). A significant positive association with 3-month mortality was observed for creatinine, urea, and NT-proBNP (Table 3). Concentrations of large HDL particles were not significantly associated with 3-month mortality (Table 3). Importantly, the concentrations of both total and small HDL particles remained significantly associated with 3-month mortality after adjusting for age and sex, as well as for clinical and laboratory parameters that showed a significant association with 3-month mortality in the univariate analyses, taking multicollinearity into account (Table 4).

## Discussion

Accurate prognostic biomarkers are critical for risk assessment, appropriate therapeutic intervention, and overall management of HF. The present study shows for the first time that a low concentration of circulating small HDL particles is associated with 3-month mortality of AHF patients. Interestingly, only the concentrations of small HDL particles were associated with mortality, whereas the concentrations of large HDL particles and HDL-cholesterol were not.

Our results appear to be in line with recent studies showing that elevated concentrations of HDL particles were associated with a reduced risk of coronary heart disease (CHD) as well as with reduced CHD mortality [reviewed in^21^]. However, as mentioned above, no single study so far has examined the role of HDL particle concentrations on mortality in AHF. Previous studies have clearly shown that cardioprotective activities of HDL, including cholesterol efflux capacity, anti-inflammatory, anti-apoptotic, as well as endothelial protective capabilities, depend on specific HDL particle characteristics that are not well represented by the HDL-cholesterol content (a measure of larger, more cholesterol-rich HDL subclasses)^21^,^22^. The results of the present study appear to be in agreement with the concept of a strong structure-function relationship of HDL particles.

Our results clearly demonstrate that circulating HDL particle concentrations, but not the levels of HDL-cholesterol, predict 3-month mortality of AHF patients. Interestingly, serum concentrations of total and small HDL particles, but not of HDL-cholesterol, were positively correlated with the albumin level, a marker of nutritional status and liver biosynthetic capacity, and negatively correlated with NT-proBNP, a marker of HF severity (Table 2). This difference between small HDL particles and HDL-cholesterol regarding their sensitivity to the AHF pathophysiology could be explained by the fact that circulating HDL-cholesterol levels mainly reflect the concentrations of large (cholesterol-rich) HDL particles. Our observation that neither HDL-cholesterol nor the concentrations of large HDL particles were correlated with albumin or NT-proBNP levels (Table 2) is in full agreement with that notion.

In line with previous studies^21^, small HDL particles constituted the majority of total HDL particles (Table 1). In the present study, the concentration of small HDL particles differed markedly from that of the less abundant large HDL particles, regarding their associations with serum albumin, the markers of renal function, urea, and GFR, as well as with the inflammatory marker CRP or with NT-proBNP (Table 2). A previous study showed that adiposity affects lipoprotein size and subclass concentrations in the general population^23^. We also observed that the concentrations of large, but not small, HDL particles were negatively correlated with the body mass index (Table 2). Strikingly, we found no correlation between the serum levels of small and large HDL particles (ρ = −0.16; p = 0.132). This observation suggests that in AHF patients the metabolism of small and large HDL particles is not linked. Our results are in agreement with a recent metabolic study showing that the liver directly secrets HDL particles of different sizes into the circulation, whereas the interconversion of circulating HDL particles appears to be of minor importance^24^.

Circulating HDL particles may exert direct positive effect on the failing heart. HDL has been shown to attenuate apoptosis, improve cell survival, preserve mitochondrial function, attenuate oxidative stress^25^,^26^, as well as to promote glucose uptake by cardiomyocytes^27^. Furthermore, by inducing the expression and activity of endothelial nitric oxide synthase, HDL increases vascular NO bioavailability^5^, thereby promoting vasorelaxation of the coronary arteries and, in turn, perfusion of the failing heart. By attenuation of the endothelial inflammatory response^3^, as well as by endotoxin-binding and neutralization, HDL may ameliorate the chronic inflammatory status in HF^28^. In line with this, we found a negative correlation of HDL particle concentrations and CRP in our AHF cohort (Table 2).

Based on our results, we conclude that serum concentrations of total and small HDL particles are strong and independent predictors of 3-month mortality in AHF patients.

Because the limited number of participants in this monocentric study may have affected the statistical power of our analyses, further large studies are needed to confirm our results.

## Methods

### Study design and patients

The study was designed as a prospective, single-center study. It included hospitalized adult Caucasian consecutive patients with AHF. The Ethics Committee of the University Hospital Centre Sisters of Charity, Zagreb, Croatia as well as the Ethics Committee of the Medical University of Graz approved the study. Written informed consent was obtained in compliance with Good Clinical Practice, and the *Declaration of Helsinki*^29^. In total, 152 patients were enrolled from November 2013 to February 2015. The patients were classified according to the European Society of Cardiology (ESC) and the American College of Cardiology Foundation/American Heart Association (ACCF/AHA) Guidelines for HF^30^,^31^,^32^. All patients were treated according to the ESC Guidelines for AHF^31^,^32^. Hypertension was diagnosed according to the ESC criteria^33^. Diabetes was diagnosed in patients with dietary treatment, antidiabetic medication or current fasting plasma glucose levels higher than 7.0 mmol/L^34^. Hypercholesterolemia was defined as LDL-cholesterol levels higher than 3.5 mmol/L, or taking a lipid-lowering drug and hypertriglyceridemia was defined as triglyceride levels higher than 1.7 mmol/L. Patients with severe renal failure (serum creatinine ≥400 mmol/L) were not included in the study.

### Laboratory assays

Blood samples were obtained at admission to the hospital. The blood was collected in 6 mL tubes, VACUETTE^®^ Z Serum Clot Activator (Greiner Bio-one GmbH, Kremsmuenster, Austria). Beckman Coulter instrument AU 2700, 2007 (Brea, CA, SAD) and Architect c8000, Abbott 2013 (Chicago, IL, SAD) were used for analysis of serum albumin, creatinine, urea, CRP, total plasma cholesterol, LDL-cholesterol, HDL-cholesterol, and triglycerides. GFR was calculated as previously described^35^. Electrochemiluminescence immunoassay with Elecsys e411 (Roche Diagnostics GmbH, Mannheim, Germany) was used for NT-proBNP quantification.

### Lipoprotein profiling by NMR spectroscopy

The lipoprotein profiles of 138 serum samples were available for analysis using the *AXINON^®^ lipoFIT^®^-*S100 test system (Numares Health, Regensburg, Germany) as described previously^36^. In brief, serum (630 μl) was gently mixed with 70 μl of an internal standard (with reference substances, NaN_3_ and D_2_O). From this solution 600 μl were transferred into 5-mm NMR tubes, followed by the recording of NMR spectra at a temperature of 310 K on a shielded 600 MHz Bruker Avance III HD spectrometer with a 5-mm triple resonance TXI probe head including a deuterium lock channel and a z-grade coil. Only samples and their spectra which met a defined set of quality criteria were analyzed, in order to ensure the data quality. Lipoprotein particle concentrations and mean sizes reported are calibrated to an NMR-based lipoprotein profiling method^37^. HDL particles with a diameter smaller than 8.8 nm were designated small, and those with a diameter of 8.8 nm or more were considered large^38^. The number (n) of samples, which were analyzed for a particular lipoprotein parameter, is indicated in Table 2.

### Statistical analysis

Categorical data are shown as absolute and relative frequencies; continuous data are shown as median and range from minimum to maximum. Correlations between various laboratory/clinical parameters and certain lipid parameters, including concentrations of HDL particles and HDL-cholesterol, were determined by Spearman correlation. Univariate logistic regression analyses was used to examine the impact of various clinical and laboratory parameters on 3-month mortality. To further assess the predictive ability of the total and small HDL particle concentrations, we adjusted for age, gender, mean arterial pressure (MAP), LDL-cholesterol, GFR, urea, and NT-proBNP in a multivariate model. Results were expressed as odds ratio (OR) and the respective 95% confidence interval (CI) per standard deviation (SD) increase. Variance inflation factor was used to assess multicollinearity among covariates in the multivariate model. R version 3.2.2. was used to analyse data.

## Additional Information

**How to cite this article:** Potočnjak, I. *et al*. Serum Concentration of HDL Particles Predicts Mortality in Acute Heart Failure Patients. *Sci. Rep.* **7**, 46642; doi: 10.1038/srep46642 (2017).

**Publisher's note:** Springer Nature remains neutral with regard to jurisdictional claims in published maps and institutional affiliations.

## Figures and Tables

**Table 1:** Laboratory parameters.

Variable	n	
Total cholesterol (mmol/L)	152	3.8 [1.7–9.1]
LDL-c (mmol/L)	152	2.3 [0.8–6.3]
HDL-c (mmol/L)	152	1.0 [0.3–3.6]
Triglycerides (mmol/L)	152	1.1 [0.5–4.3]
Large VLDL-p (nmol/L)	76	2.4 [1.5–10.6]
Total LDL-p (μmol/L)	135	1.1 [0.4–2.5]
Large LDL-p (μmol/L)	126	0.7 [0.3–2.1]
Small LDL-p (μmol/L)	132	0.5 [0.2–1.4]
Total HDL-p (μmol/L)	135	21.0 [3.7–37.5]
Large HDL-p (μmol/L)	108	5.7 [2.8–14.9]
Small HDL-p (μmol/L)	115	17.0 [6.3–34.2]
VLDL size (nm)	135	47.1 [37.1–52.3]
LDL size (nm)	135	21.2 [20.2–22.7]
HDL size (nm)	135	9.2 [8.3–10.6]
CRP (μg/mL)	150	9.4 [0.2–247.4]
Albumin (g/L)	149	40.0 [21.0–72.0]
Protein (g/L)	149	68.0 [31.0–87.0]
Urea (mmol/L)	151	8.0 [3.0–64.0]
Creatinine (μmol/L)	151	106.0 [53.0–273.0]
GFR (mL/min/1.73 m^2^)	151	50.9 [15.0–105.7]
NT-proBNP (pg/mL)	141	9570.0 [171.0–70000.0]

Results are presented as median, minimum and maximum.

CRP = C-reactive protein; GFR = glomerular filtration rate; HDL-c = high-density lipoprotein cholesterol; HDL-p = HDL particle concentration; LDL-c = low-density lipoprotein cholesterol; LDL-p = LDL particle concentration; n = number of samples in which particular parameter was analysed; NT-proBNP = N-terminal pro-Brain Natriuretic Peptide; VLDL-p = very low-density lipoprotein particle concentration.

**Table 2:** Correlation analyses of HDL particle- and HDL cholesterol- concentrations with laboratory and clinical parameters.

		Large HDL-p (μmol/L)	Small HDL-p (μmol/L)	Total HDL-p (μmol/L)	HDL-c (mmol/L)
**Albumin (g/L)**	ρ	−0.12	0.23	0.28	0.02
	p	0.223	**0.012**	**0.001**	0.787
	n	105	114	132	149
**Urea (mmol/L)**	ρ	−0.05	−0.22	−0.31	−0.24
	p	0.608	**0.019**	**<0.001**	**0.003**
	n	107	114	134	151
**GFR (mL/min/1.73** **m**^**2**^)	ρ	0.08	0.27	0.25	0.17
	p	0.398	**0.003**	**0.004**	**0.037**
	n	107	114	134	151
**NT-proBNP (pg/mL)**	ρ	0.07	−0.31	−0.44	−0.16
	p	0.478	**0.001**	**<0.001**	**0.061**
	n	104	11	129	141
**CRP (μg/mL)**	ρ	−0.12	−0.28	−0.43	−0.28
	p	0.231	**0.003**	**<0.001**	**<0.001**
	n	106	113	133	150
**Body weight (kg)**	ρ	−0.29	0.12	0.1	−0.12
	p	**0.002**	0.197	0.233	0.138
	n	108	115	135	152
**BMI (kg/m**^**2**^)	ρ	−0.28	0.18	0.15	−0.11
	p	**0.003**	0.056	0.089	0.197
	n	108	115	135	152

Data are presented as Spearman correlation coefficient, p-value and number of samples; significant associations are depicted in bold.

CRP = C-reactive protein; GFR = glomerular filtration rate; HDL-c = high-density lipoprotein cholesterol; HDL-p = HDL particle concentration; n = number of samples in which correlation was analysed; NT-proBNP = N-terminal pro-Brain Natriuretic Peptide; p = p value; ρ = Spearman correlation coefficient.

**Table 3:** Univariate logistic regression analysis of clinical and laboratory parameters, and 3-month mortality.

Variable	Category	OR (95% CI) per 1 SD	p-value	Events
Age (years)		1.37 (0.94–2.08)	0.114	40/146
Sex	male	Ref.		16/70
	female	1.56 (0.75–3.30)	0.239	24/76
BMI (kg/m^2^)		0.70 (0.47–1.02)	0.074	40/146
Hypertension	no	Ref.		7/16
	yes	0.44 (0.15–1.31)	0.128	33/130
Type 2 Diabetes Mellitus	no	Ref.		21/69
	yes	0.76 (0.36–1.58)	0.465	19/76
MAP (mmHg)		0.56 (0.36–0.84)	**0.007**	40/146
NYHA class	2 and 3	Ref		20/90
	4	1.94 (0.93–4.09)	0.078	20/56
EF (%)*	>40	Ref		14/71
	≤40	1.44 (0.65–3.26)	0.373	17/65
Total cholesterol (mmol/L)		0.53 (0.33–0.80)	**0.005**	40/146
LDL-c (mmol/L)		0.56 (0.34–0.85)	**0.011**	40/146
HDL-c (mmol/L)		0.69 (0.43–1.05)	0.107	40/146
Triglycerides (mmol/L)		0.74 (0.46–1.10)	0.172	40/146
Large VLDL-p (nmol/L)		0.43 (0.15–0.91)	0.066	20/74
Total LDL-p (μmol/L)		0.68 (0.44–1.02)	0.075	37/130
Large LDL-p (μmol/L)		0.63 (0.38–0.98)	0.057	34/121
Small LDL-p (μmol/L)		0.89 (0.58–1.30)	0.561	36/127
Total HDL-p (μmol/L)		0.38 (0.23–0.60)	**<0.001**	37/130
Large HDL-p (μmol/L)		1.22 (0.79–1.88)	0.353	30/103
Small HDL-p (μmol/L)		0.35 (0.19–0.60)	**<0.001**	27/110
GFR (ml/min/1.73 m^2^)		0.60 (0.39–0.88)	**0.012**	39/145
Creatinine (mol/L)		1.44 (1.02–2.06)	**0.039**	39/145
Urea (mmol/L)		1.61 (1.13–2.39)	**0.013**	39/145
NT-proBNP (pg/mL)		1.92 (1.32–2.93)	**0.001**	38/135
CRP (μg/mL)		1.18 (0.83–1.68)	0.341	40/145

For continous variables ORs are presented per standard deviation increment. Significant associations are depicted in bold. BMI = Body Mass Index; CI = confidence interval; CRP = C-reactive protein; EF = Ejection Fraction; Events = number of events/total number of patients in category; GFR = glomerular filtration rate; HDL-c = high density lipoprotein cholesterol; HDL-p = HDL particle concentration; LDL-c = low-density lipoprotein cholesterol; LDL-p = large LDL particle concentration; n = number of samples in which particular parameter was analysed; MAP = Mean Arterial Pressure; NT-proBNP = N-terminal pro-Brain Natriuretic Peptide; NYHA = New York Heart Association Functional Classification; OR = odds ratio; Ref. = reference category; SD = standard deviation; VLDL-p = very low-density lipoprotein particle concentration.

**Table 4:** Multivariate logistic regression analysis of 3-month mortality for total and small HDL particle concentrations.

	OR (95% CI) per 1 SD	p-value	Events
**Total HDL-p (μmol/L)**	0.43 (0.21–0.83)	**0.016**	35/123
**Small HDL-p (μmol/L)**	0.40 (0.16–0.88)	**0.031**	25/104

The model was adjusted for age, sex, MAP, LDL cholesterol, GFR, urea, and NT-proBNP.

CI = confidence interval; Events = number of events/total number of patients in category; GFR = glomerular filtration rate; HDL-p = HDL particle concentration; LDL = low-density lipoprotein; MAP = Mean Arterial Pressure; NT-proBNP = N-terminal pro-Brain Natriuretic Peptide; OR = odds ratio; SD = standard deviation.
